# Plant pathogen‐mediated rapid acclimation of a host‐specialized aphid to a non‐host plant

**DOI:** 10.1002/ece3.8209

**Published:** 2021-10-11

**Authors:** Farhan Ali, Xiaoyue Hu, Duoqi Wang, Fengying Yang, Hao Guo, Yongmo Wang

**Affiliations:** ^1^ Hubei Insect Resources Utilization and Sustainable Pest Management Key Laboratory College of Plant Science and Technology Huazhong Agricultural University Wuhan China

**Keywords:** host race, host range expansion, host shift, host‐plant specialization, phytopathogen‐mediated host adaptation, plant–parasite interaction, polyphagous aphid

## Abstract

Polyphagous aphids often consist of host‐specialized lineages, which have greater fitness on their native hosts than on others. The underlying causes are important for understanding of the evolution of diet breadth and host shift of aphids. The cotton‐melon aphid *Aphis gossypii* Glover is extremely polyphagous with many strict host‐specialized lineages. Whether and how the lineage specialized on the primary host hibiscus shifts to the secondary host cucumber remains elusive. We found that the hibiscus‐specialized lineage suffered high mortality and gave birth to very few nymphs developing into yellow dwarfs on fresh cucumber leaves, and did not inflict any damage symptoms on cucumber plants. The poor performance did not improve with prolonged exposure to cucumber; however, it did significantly improve when the cucumber leaves were pre‐infected with a biotrophic phytopathogen *Pseudoperonospora cubensis*. More importantly, the hibiscus‐specialized lineage with two‐generation feeding experience on pre‐infected cucumber leaves performed as well as the cucumber‐specialized lineage did on fresh cucumber leaves, and inflicted typical damage symptoms on intact cucumber plants. Electrical penetration graph (EPG) indicated that the hibiscus‐specialized lineage did not ingest phloem sap from fresh cucumber leaves but succeeded in ingesting phloem sap from pre‐infected cucumber leaves, which explained the performance improvement of the hibiscus‐specialized lineage on pre‐infected cucumber leaves. This study revealed a new pathway for the hibiscus‐specialized lineage to quickly acclimate to cucumber under the assistance of the phytopathogen. We considered that the short feeding experience on pre‐infected cucumber may activate expression of effector genes that are related to specific host utilization. We suggest to identify host‐specific effectors by comparing proteomes or/and transcriptomes of the hibiscus‐specialized lineage before and after acclimating to cucumber.

## Graphical abstract

The hibiscus‐specialized *Aphis gossypii* cannot use cucumber as host plant but performed well on cucumber that has been pre‐infected with the phytopathogen Pseudoperonospora cubensis; a short feeding experience on the pre‐infected cucumber enables the hibiscus‐specialized *A*. *gossypii* to fully adapt to fresh cucumber.

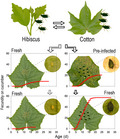

## 1 INTRODUCTION

Theoretically, insect herbivores should evolve toward being dietary generalists because using multiple host plants is advantageous to using one host plant; however, most of them are dietary specialists (Forister et al., 2012; Jaenike, 1990). Even in species with a broad diet breadth (polyphagous species), host‐specialized populations or host races are frequently observed in nature (Drès & Mallet, 2002). Host specialization, generally result from a performance trade‐off, is characterized by preferring or performing much better on native hosts than on novel hosts (Joshi & Thompson, 1995). A genetic basis for performance trade‐offs has been reported in many insect herbivores (Booth et al., 2015; Hawthorne & Via, 2001; Peccoud et al., 2014; Powell et al., 2014; StiremanIII et al., 2005), explaining why gene flow is restricted among host‐specialized populations of these species. What determines host specialization and whether it can be altered in the short term is important for understanding the evolution of insect diet breadth (Forister et al., 2015; Hardy et al., 2020). During the course of crop domestication, some insect herbivores successfully shifted from wild plants to domesticated crops to become major pests (Chen et al., 2015; Simon & Peccoud, 2018). Therefore, the understanding of host shift and host specialization is also important for managing insect pests in agricultural ecosystems.

The cotton‐melon aphid, *Aphis gossypii* Glover, is extremely polyphagous, damaging more than 900 plant species in 116 plant families, including many important horticultural crops, such as melon, cucumber, eggplant, potato, cotton, okra, and chrysanthemum (Blackman & Eastop, 2000). Previous studies reported that host‐associated populations of *A*. *gossypii* exhibited high fidelity to their native host plants (Guldemond et al., 1994; Liu et al., 2002; Wool et al., 1995), which was highlighted in reciprocal host transfer experiments between different host‐associated lineages. For example, clones (a clone referring to a cohort aphids derived from a parthenogenetic aphid with specific genotype) collected from cucumber were unable to establish population on chrysanthemum and vice versa (Guldemond et al., 1994); clones collected from hibiscus cannot survive when they were transferred to cucumber (Carletto et al., 2009; Liu et al., 2002, 2004; Satar et al., 2013); clones collected from cotton cannot survive on cucumber and pumpkin, and those from cucumber cannot survive on cotton (Liu et al., 2002, 2004; Najar‐Rodríguez et al., 2009). However, clones collected from hibiscus were able to use cotton as a suitable host plant and vice versa (Liu et al., 2002, 2004; Najar‐Rodríguez et al., 2009). In fact, the hibiscus‐ and the cotton‐associated clones belong to the same host‐specialized lineage (Wang et al., 2016). In addition to performance trade‐offs across hosts, some studies detected genetic differences among host‐associated populations of *A*. *gossypii* (Carletto et al., 2009; Charaabi et al., 2008; Vanlerberghe‐Masutti & Chavigny, 1998; Wang et al., 2017), indicating the evolution of host races in this aphid.

In cold‐winter areas, such as north China, *A*. *gossypii* fulfills holocyclic life cycle, reproducing asexually on various secondary hosts and returning to primary hosts once a year for sexual reproduction (producing cold‐resistant eggs) (Blackman & Eastop, 2000). Hibiscus is the main primary host (overwintering host) of *A*. *gossypii* in north China. Parthenogenetic aphids cannot produce cold‐resistant eggs nor survive cold winters on secondary hosts (Gilabert et al., 2009). So, *A*. *gossypii* on various secondary hosts must return to primary hosts to lay eggs before winter comes. As described above, the hibiscus‐associated lineage of *A*. *gossypii* exhibits a high degree of specialization on hibiscus and cannot survive on some secondary hosts, such as cucumber. Although direct shift proved impossible, it is not ruled out that the hibiscus‐associated lineage can shift indirectly from hibiscus to cucumber.

To address this issue, we conducted field observations over several years in a garden where cucumber, cotton, and hibiscus were co‐planted. *Aphis gossypii* infestation was not observed when the cucumber plants were young and healthy, even though the adjacent cotton and hibiscus plants harbored a large population of *A*. *gossypii*; however, infestation occurred in cucumbers that were infected with certain phytopathogens, generally initiating from the lower leaves of the cucumber (Y. Wang, personal observation). We suspected that the *A*. *gossypii* infesting the cucumber came from hibiscus or cotton plants in the garden. Recently, there has been significant progress in the understanding of molecular interactions between parasitic organisms and host plants, such as aphids (Furch et al., 2015), nematodes (Yang et al., 2019), and phytopathogens (Yin et al., 2019). Successful infection or infestation requires that the parasitic organisms can suppress host‐plant defense responses (Will et al., 2007), generally via molecular effectors targeting specific plant receptors (Rodriguez et al., 2017). A preprint article reported that molecular effectors of the oomycete *Phytophthora capsici* and the aphid *Myzus persicae* targeted the same immune regulator of *Arabidopsis* (Liu et al., 2020), indicating that different plant parasitic organisms can assist each other in suppressing defenses of the common host plant. Although the hibiscus‐specialized lineage of *A*. *gossypii* cannot use fresh cucumber as host, they may be able to colonize cucumber when the defenses of the cucumber have been suppressed by certain plant pathogens. Based on our field observations and the literature, we hypothesized that the hibiscus‐specialized lineage of *A*. *gossypii* can colonize cucumber under the assistance of certain plant pathogens.

To test this hypothesis, we first tested the degree of host specialization of *A*. *gossypii* collected from hibiscus and cucumber by reciprocal host transfer experiments. Then, we chose the biotrophic phytopathogen *Pseudoperonospora cubensis* to infect cucumber leaves and compared the fitness of the hibiscus‐specialized lineage on pre‐infected and fresh cucumber leaves using life table methods. Next, we transferred the hibiscus‐specialized lineage with feeding experience on pre‐infected cucumber leaves to intact cucumber plants, to compare the damage symptoms inflicted by it with that inflicted by the cucumber‐specialized lineage. Finally, we monitored aphid piercing and sucking behaviors to understand why the fitness of the hibiscus‐specialized lineage on pre‐infected cucumber leaves was different from that on fresh cucumber leaves. We aimed to reveal a new pathway for host‐specialized aphids to rapidly acclimate to new host plants and provide new insights into the evolution of insect diet breadth.

## 2 MATERIAL AND METHODS

### 2.1 Aphid materials

We collected wingless parthenogenetic individuals of *A*. *gossypii* from hibiscus (*Hibiscus syriacus*) and cucumber (*Cucumis sativus*) from Baoding (38°53′N; 115°28′E) in the spring of 2015. The host‐associated aphid samples were considered as the hibiscus‐specialized (HI) lineage and the cucumber‐specialized (CU) lineages, respectively. Baoding, located in the region of northern China, has a typical temperate continental climate, with lowest temperatures below −10°C in winter months, during which parthenogenetic aphids cannot survive. Each lineage includes four clones whose collection sites were more than 1 km apart. The clones of the CU lineage were reared separately in nylon net cages using cucumber seedlings at three‐ to five‐leaf stage at 22 ± 2°C and a 16:8‐h (L/D) photoperiod. The clones of the HI lineage were reared using cotton seedlings because both hibiscus and cotton are suitable host plants of the HI lineage (Carletto et al., 2009; Liu et al., 2002, 2004; Najar‐Rodríguez et al., 2009; Wang et al., 2016). The aphid materials were transferred to new plants every 2 weeks to prevent overcrowding and host‐plant deteriorating.

### 2.2 Host‐plant specialization test

Host‐plant specialization of the HI and CU lineages was tested using reciprocal host transfer experiments. A 7‐day‐old detached leaf of cucumber or cotton was placed in a 12‐cm Petri dish filled with 30 ml 1.5% agar gel. The leaf petiole was wrapped with wet cotton wool. Five wingless adults were introduced into each Petri dish and were removed after 12 h, leaving less than 20 new nymphs in each leaf in the Petri dish. The Petri dishes were wrapped in transparent nylon net bags (0.16‐mm mesh size) and were placed in an artificial climate chamber at 22 ± 2 °C with a 16:8‐h (L/D) photoperiod. Four treatments were set as follows: the HI lineage on cotton and cucumber, the CU lineage on cotton and cucumber. Each treatment included one clone and was replicated three times (three Petri dishes). The newborn nymphs were observed for survival, development, and reproduction every day until they died. When the newborn nymphs reached adulthood (6–7 day old), they were photographed under a binocular microscope (Nikon). Their offspring were removed from the Petri dishes after being counted every day. All the four clones of the HI or CU lineage were tested for host‐plant specialization in our preliminary qualitative experiments, and all of them exhibited high degree of specialization to their native hosts. Only one clone in each lineage was used in the subsequent quantitative test of host‐plant specialization and in the following experiments.

### 2.3 Life table of the HI lineage on fresh and pre‐infected cucumber

In order to test our hypothesis that the hibiscus‐specialized lineage of *A*. *gossypii* can colonize cucumber under the assistance of certain plant pathogens, we artificially infected cucumber leaves with *P*. *cubensis* and compared the fitness of the HI lineage on pre‐infected and fresh cucumber leaves for three generations using the life table method. We chose *P*. *cubensis* (cucumber downy mildew) to pre‐infect cucumber leaves because our field observations found that *A*. *gossypii* infestation mostly started from lower leaves that were infected with *P*. *cubensis*. Like aphids, *P*. *cubensis*, a biotrophic phytopathogen, steals nutrients from living plant tissues (Hahn & Mendgen, 2001), and it may have similar mechanisms in suppressing plant defenses as aphids. We collected *P*. *cubensis* from cucumber plants in a greenhouse in Wuhan, China. To identify the pathogen, DNA was extracted from the pathogen samples that were prepared by the method proposed by Cai et al. (2008) and amplified for the partial sequence of 18S rDNA using ITS1 (5′‐TCCGTAGGTGAACCTGCGG‐3′) and ITS4 (5′‐TCCTCCGCTTATTGATATGC‐3′) primers described by White et al. (1990). The PCR protocol followed that described by Cai et al. (2008). A BLAST similarity search indicated the amplicon has more than 99% similarity to the first 20 matching sequences that were registered as *P*. *cubensis* (see Figure S1). Spores of *P*. *cubensis* were washed down from the field‐collected leaves using distilled water to make a suspension of approximately 10^5^ spores per milliliter. Concentration of spores was determined by microscopic counting in a hemocytometer. The suspension was sprayed on fresh cucumber leaves, and the leaves were then placed in Petri dishes filled with 1.5% agar gel. The inoculated leaves were incubated at 22 ± 2°C in constant darkness for 24 h and then were moved to a 16:8‐h (L/D) photoperiod under the same temperature. Symptoms of infection appeared within 6–7 days. The infected leaves were used as pre‐infected leaves. The leaves that underwent the same process except for the phytopathogen inoculation were used as fresh leaves.

We established six life tables by the following methods. Wingless adult aphids of the HI lineage were introduced into fresh and pre‐infected cucumber leaves (five aphids per leaf) in a Petri dish (ϕ14 cm) filled with 30 ml 1.5% agar gel, and the adults were removed after 12 h, leaving five newborn nymphs (F1). The nymphs were observed for survival and reproduction every day until they died to construct the life table ‘HI_fre_F1’ and ‘HI_infe_F1’, respectively. The offspring of F1 (F2) were removed from Petri dishes after being counted. Some F2 nymphs were transferred to leaves with similar treatment to those that F1 were born on, to produce F3. Newborn F3 nymphs were transferred to leaves (five nymphs per leaf) with similar treatment to those that F2 were born on to construct the life table ‘HI_fre_F3’ and ‘HI_infe_F3’. In order to test how the HI lineage with feeding experience on pre‐infected cucumber leaves performs on fresh cucumber leaves, newborn F3 nymphs from the pre‐infection treatment were transferred to fresh cucumber leaves (five nymphs per leaf) to construct the life table ‘HI‐acclimated_fre’, with the life table of the CU lineage on fresh cucumber named ‘CU_fre’ as a control. Detailed information about the life table settings was shown in Table 1. Each life table replicated 50 aphids (10 Petri dishes, each containing five aphids).

**TABLE 1:** Life table settings of the hibiscus‐specialized lineage on fresh and pre‐infected cucumber leaves with *Pseudoperonospora cubensis*

Life table code^a^	Aphid lineage^b^	Status of cucumber leaves	Experience of the aphids before the experiment
HI_fre_F1	HI	Fresh	Cultured on cotton for multiple generations
HI_infe_F1	HI	Pre‐infected	Cultured on cotton for multiple generations
HI_fre_F3	HI	Fresh	Cultured on fresh cucumber for two generations
HI_infe_F3	HI	Pre‐infected	Cultured on pre‐infected cucumber for two generations
HI‐acclimated_fre	HI	Fresh	Cultured on pre‐infected cucumber for two generations
CU_fre	CU	Fresh	Cultured on cucumber for multiple generations

^a^ Aphids at the beginning of each experiment were newborn nymphs (1st instar).

^b^ HI and CU indicate the hibiscus‐specialized and cucumber‐specialized lineages, respectively.

### 2.4 Body development of the HI lineage on fresh and pre‐infected cucumber

The body size and color of *A*. *gossypii* varies greatly on suitable and unsuitable host plants (Watt & Hales, 1996). In order to test whether pre‐infected cucumber leaves were more suitable for the HI lineage than fresh cucumber leaves, we measured the body size of aphids grown in the six life table experiments. Nymphs of *A*. *gossypii* develop into adults and reach the maximum body size at the age of 6–7 days. Therefore, the aphids were measured for the body length and width at the age of 7 days under a binocular microscope (Nikon). Thirty aphids were measured in each treatment.

### 2.5 Reproduction and damage symptoms of the acclimated HI lineage on cucumber plants

Detached leaves may be different from intact plants in defenses against aphid attacks. To test how the HI lineage with feeding experience on pre‐infected cucumber leaves for two generations (the acclimated HI lineage) performed on intact cucumber plants, we introduced newborn nymphs of the acclimated HI lineage to intact three‐leaf stage cucumber plants. Newborn nymphs of the HI lineage with no experience on pre‐infected cucumber acted as a negative control, and newborn nymphs of the CU lineage acted as a positive control. Each treatment included five cucumber plants (five replicates), and each plant was introduced 20 nymphs. The plants were cultured at 22 ± 2°C and a 16:8‐h (L/D) photoperiod. Number of aphids per plant and damage symptoms were recorded after 2 weeks.

### 2.6 Feeding behaviors of the HI lineage on fresh and pre‐infected cucumber

In order to determine the reasons for the performance differences on fresh and pre‐infected cucumber leaves, the piercing and sucking behaviors of the HI lineage on fresh and pre‐infected cucumber leaves were monitored using an electrical penetration graph (EPG) system (Tjallingii, 1988). Cucumber leaves and aphids were made parts of an electrical circuit in this system. An electrode attached to a gold wire (diameter 20 μm) was inserted into the petiole of a leaf, and the other end of the gold wire was attached to dorsum an aphid using electric‐conducting glue. When the aphid started probing the plant cells, the circuit was closed, and electrical signals were generated. EPG signals (referred to as waveforms) result from voltage fluctuations correlating to the stylet locations within leaf tissues (Tjallingii & Esch, 1993). Five‐day‐old aphids were used and were monitored for 6 h under 22 ± 2°C. Three treatments were set as follows: the HI lineage on fresh cucumber leaves (HI_fre), the HI lineage on pre‐infected cucumber leaves (HI_infe), and the CU lineage on fresh cucumber leaves (CU_fre). Each treatment consisted of more than 10 successful recordings. Data were analyzed using Stylet+a (v1.25; EPG Systems). Four typical waveforms were analyzed as follows: (1) C waveform, reflecting the stylet in contact with the epidermis and penetrating the epidermis and mesophyll cells (stylet pathway phase), and (2) G waveform, reflecting active sap ingestion from xylem elements (xylem ingestion phase). When the stylet reaches the plant phloem, two types of waveforms occur as follows: (3) E1 and (4) E2 waveforms. E1 is often a short phase of the stylet secreting saliva into the phloem element (phloem salivation phase). E2 is the phloem sap ingestion phase, which can last for hours or days on suitable host plants (phloem ingestion phase).

### 2.7 Statistical analysis

The log‐rank test was applied to compare survival rates in the host specialization test, and the independent samples *t*‐test was applied to compare the accumulated fecundity, using statistical software package SPSS (v19.0; IBM Corp.). The nonparametric Mann–Whitney *U*‐test was conducted for pairwise comparison of the body size and the duration of waveforms between fresh and pre‐infected treatments also using SPSS. Life table data were analyzed according to the age‐stage, two‐sex life table theory (Chi, 1988) using TWOSEX‐MSChart software (Chi, 2015). Four parameters were calculated: net reproductive rate (*R*_0_), intrinsic rate of increase (*r*), finite rate of increase (*λ*), and mean generation time (*T*). Age‐specific survival rate and fecundity were also calculated using the TWOSEX‐MSChart and visualized using SigmaPlot software (v14.0; Systat Software Inc.). The means and standard errors of the parameters were estimated using the bootstrap method with 100,000 permutations and were compared between fresh and pre‐infected treatment using a paired bootstrap test.

## 3 RESULTS

### 3.1 Host‐plant specialization of the HI and CU lineage

**FIGURE 1:**
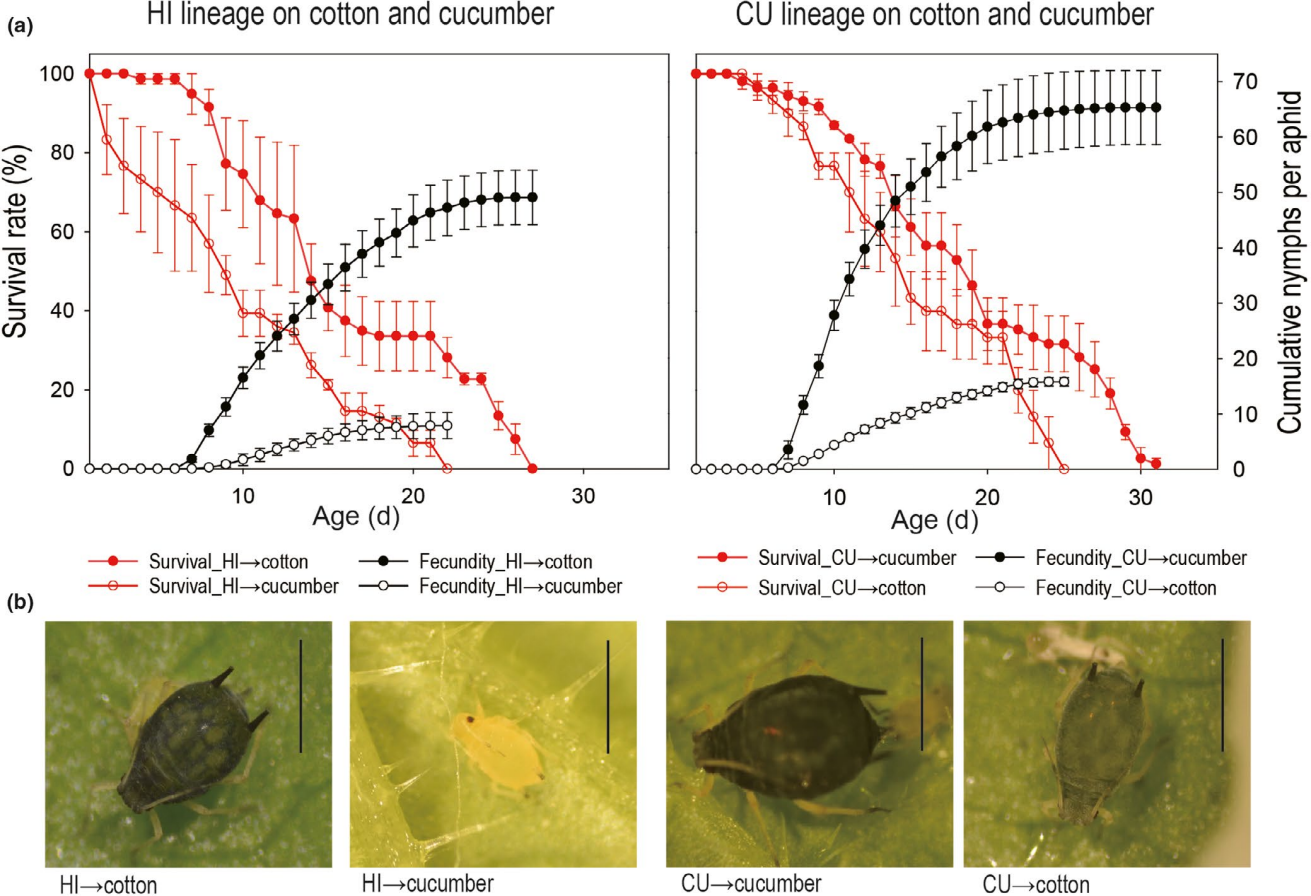
Host‐plant specialization test of the hibiscus‐specialized (HI) and the cucumber‐specialized (CU) lineages of *Aphis gossypii* used in this study. The left‐hand axis refers to the red symbols and the right‐hand axis to the black symbols. (a) Survival and fecundity of the HI lineage on cotton and cucumber; (b) Survival and fecundity of the CU lineage on cotton and cucumber; (c) Adult (7 day old) morphology of the HI and CU lineages developed on cotton and cucumber. Both cotton and hibiscus are suitable hosts of the HI lineage, so cotton was used as a substitute of hibiscus in the experiments. The error bars represent standard errors; vertical lines on the photographs indicate 1 mm

The average life span of the HI lineage was reduced by 40.59%, and reproduction was reduced by 84.10% on cucumber leaves compared to that on cotton leaves; the survival rate (log‐rank test, *p* = .008) and fecundity (*t*‐test, *p* = .002) were significantly different between the two treatments (Figure 1a). Nymphs of the HI lineage developed into yellow dwarfs on cucumber leaves, which was quite different from what they developed to on cotton leaves (Figure 1b). The life span of the CU lineage was reduced by 19.54%, and reproduction was reduced by 75.81% on cotton compared to that on cucumber; the survival rate (log‐rank test, *p* = .011) and fecundity (*t*‐test, *p* = .011) were also significantly different (Figure 1a). Adults of the CU lineage developed on cotton were smaller in body size and lighter in body color than those developed on cucumber (Figure 1b). The results verified that both lineages, especially the hibiscus‐specialized lineage, had high degree of host‐plant specialization on their respective hosts.

### 3.2 Life table parameters of the HI lineage on fresh and pre‐infected cucumber leaves

**FIGURE 2:**
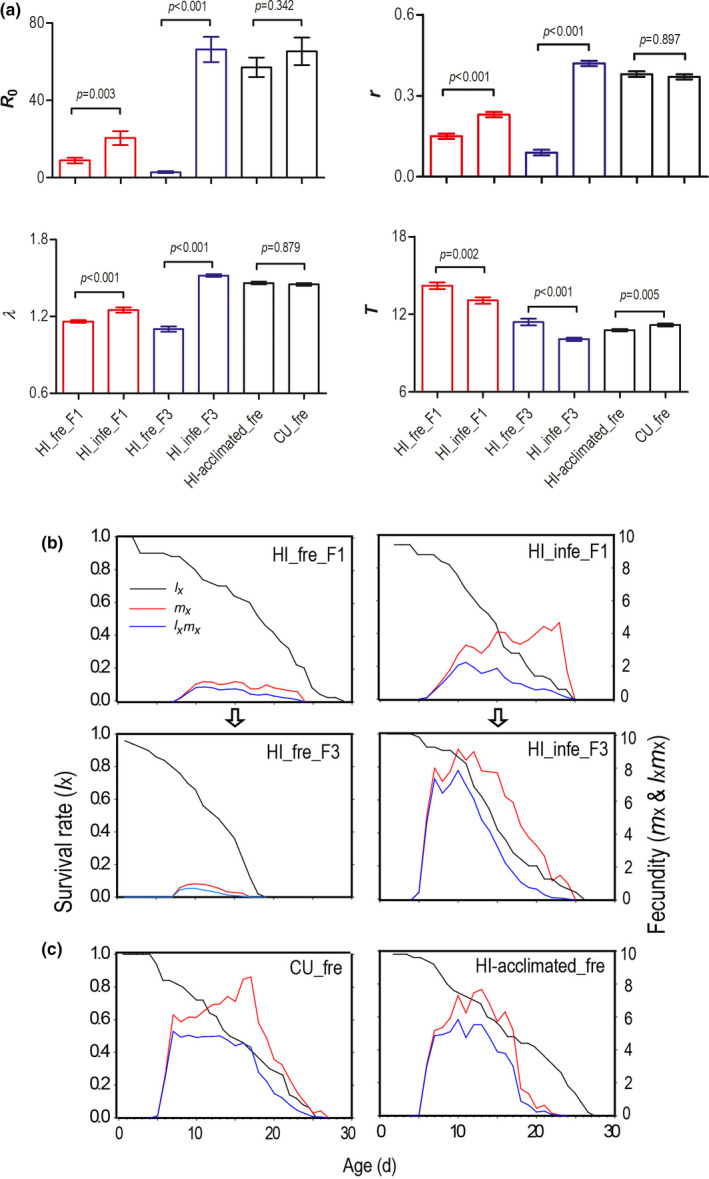
Life table analysis of the hibiscus‐specialized lineage (HI) of *Aphis gossypii* on fresh (fre) and pre‐infected (infe) cucumber leaves with *Pseudoperonospora cubensis* in three consecutive generations. The left‐hand axes refer to survival rate (*l*_x_) and the right‐hand axes to fecundity (*m*_x_ and *l*_x_ *m*_x_). (a) Life table parameters, *R*_0_ = net reproductive rate, *r* = the intrinsic rate of increase, *λ* = finite rate of increase, *T* = generation time. (b) The survival and fecundity of the HI lineage on fresh and pre‐infected cucumber leaves. (c) The survival and fecundity of the HI lineage with two‐generation feeding experience on pre‐infected cucumber (HI‐acclimated) and the cucumber‐specialized lineage (CU) on fresh cucumber leaves. Error bars represent standard error estimated by 100,000 bootstrap replicates. *p*‐values are significance levels of paired bootstrap comparisons

The HI lineage had a significantly higher reproductive rate (demonstrated by significantly higher *R*_0_, *r*, and *λ*) and a lower generation time (demonstrated by significantly lower *T*) on pre‐infected leaves than on fresh leaves in both F1 and F3 generations (Figure 2a), indicating the HI lineage had a higher fitness on pre‐infected cucumber leaves. We transferred the nymphs produced by the HI lineage that was cultured on pre‐infected cucumber leaves for two generations (HI‐acclimated) to fresh cucumber leaves, and we found that they performed as well as the CU lineage on fresh cucumber leaves, as demographic parameters were not significantly different for *R*_0_ (*p* = .342), *r* (*p* = .897) and *λ* (*p* = .879) (Figure 2a). The survival and fecundity curves also showed that the HI lineage performed far better on pre‐infected cucumber than on fresh cucumber (Figure 2b), and that the HI lineage with feeding experience on pre‐infected cucumber leaves (HI‐acclimated) performed as well as the CU lineage on fresh cucumber leaves (Figure 2c).

### 3.3 Aphid body size differences on fresh and pre‐infected cucumber leaves

**FIGURE 3:**
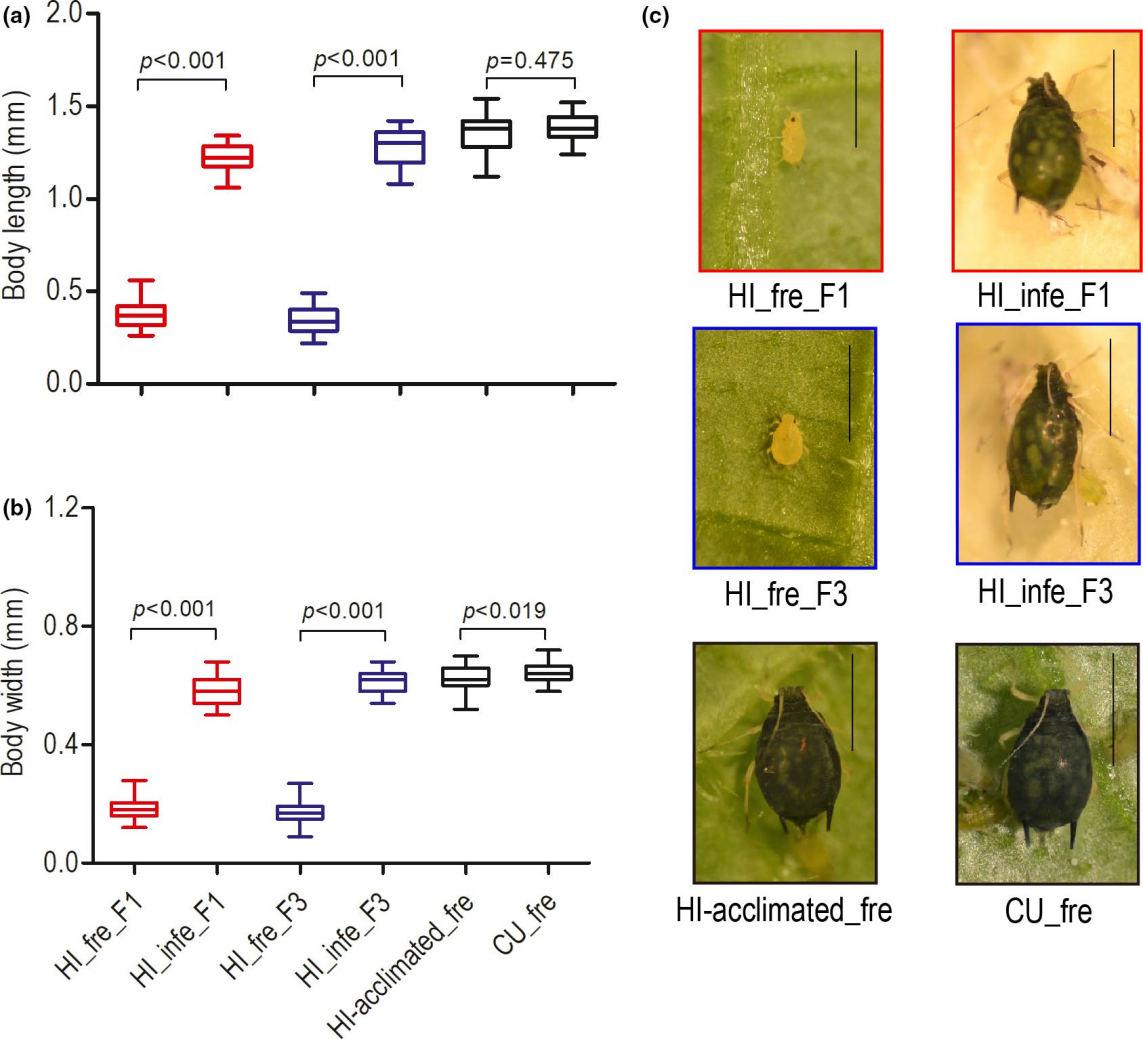
Aphid morphology of the hibiscus‐specialized lineage (HI) of *Aphis gossypii* developing on fresh and pre‐infected cucumber leaves with *Pseudoperonospora cubensis*. (a) Body length; (b) Body width; (c) The morphology of adult aphids (7 day old). Vertical whiskers attached to the boxes indicate min. and max. values. *p*‐values are significance levels of paired comparison in Mann–Whitney *U*‐test (*N* = 30). Vertical lines on right side of the photographs indicate 1 mm

The HI lineage on pre‐infected cucumber leaves developed into large green morphs in both F1 and F3, while The HI lineage cultured on fresh cucumber leaves developed into yellow dwarfs (Figure 3c). The large green morphs were significantly bigger than the yellow dwarfs in terms of body length and width (Mann–Whitney *U*‐test, *p* < .001) (Figure 3a,b). More importantly, the HI lineage with feeding experience on pre‐infected cucumber for two generations (HI‐acclimated) developed into large green morphs on fresh cucumber (Figure 3c), and those large green morphs were not different from the CU lineage adults developed on fresh cucumber in body length (Mann–Whitney *U*‐test, *p* = .475) and body color (Figure 3a,b).

### 3.4 Performance of the acclimated HI lineage on intact cucumber plant

**FIGURE 4:**
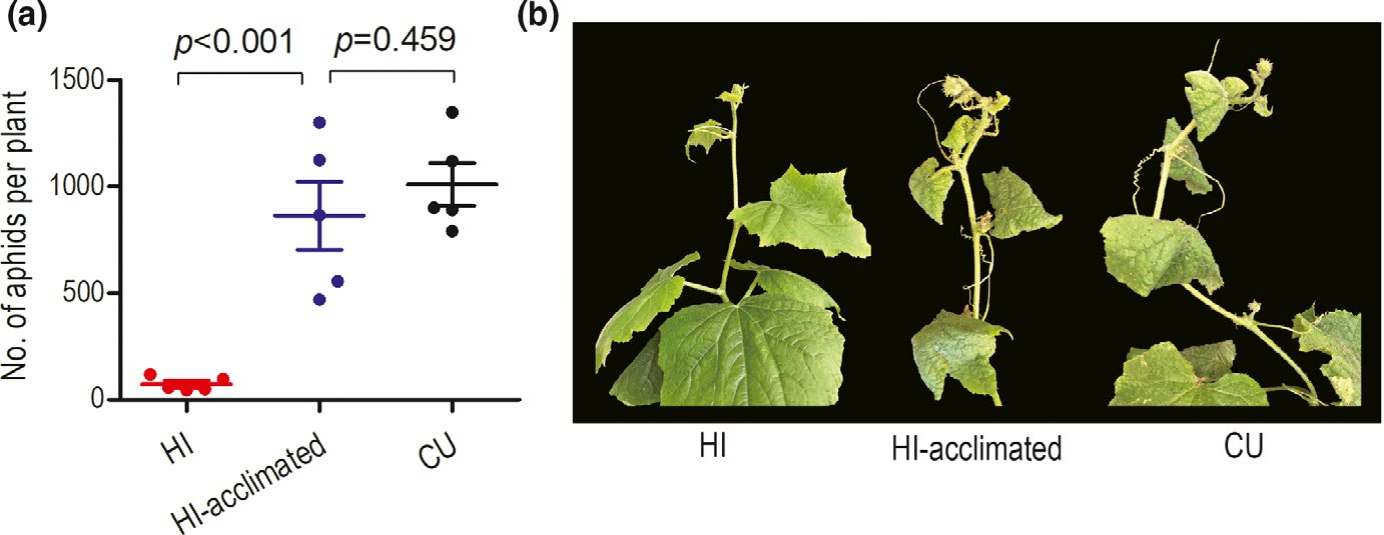
Reproduction (a) and damage symptoms (b) of the hibiscus‐specialized lineage of *Aphis gossypii* with feeding experience on pre‐infected cucumber leaves for two generations (HI‐acclimated) on intact cucumber plants. The HI lineage and the CU lineage (the cucumber‐specialized lineage) acted as negative control and positive control, respectively. The error bars represent standard errors. *p*‐values are significances of the independent samples *t*‐test. The damage symptoms showed on photographs are representative of those observed during the experiments

On intact cucumber plants, the HI lineage with feeding experience on pre‐infected cucumber for two generations (HI‐acclimated) maintained similar population growth as the CU lineage (*t*‐test, *p* = .459) and produced 11 times more nymphs than the HI lineage with no experience on pre‐infected cucumber (*t*‐test, *p* < .001) (Figure 4a). The nymphs of the acclimated HI lineage developed into large green morphs that had no discernible difference from the adults of the CU lineage developed on intact cucumber plants. The acclimated HI lineage inflicted similar damage symptoms to intact cucumber plants as the CU lineage (Figure 4b). The HI lineage with no feeding experience on pre‐infected cucumber developed into yellow dwarfs and inflicted no damage symptoms to intact cucumber plants (Figure 4b). The results indicated that the HI lineage acquired the ability to colonize intact cucumber plants during the short feeding experience on pre‐infected cucumber leaves.

### 3.5 Piercing and sucking behaviors

**FIGURE 5:**
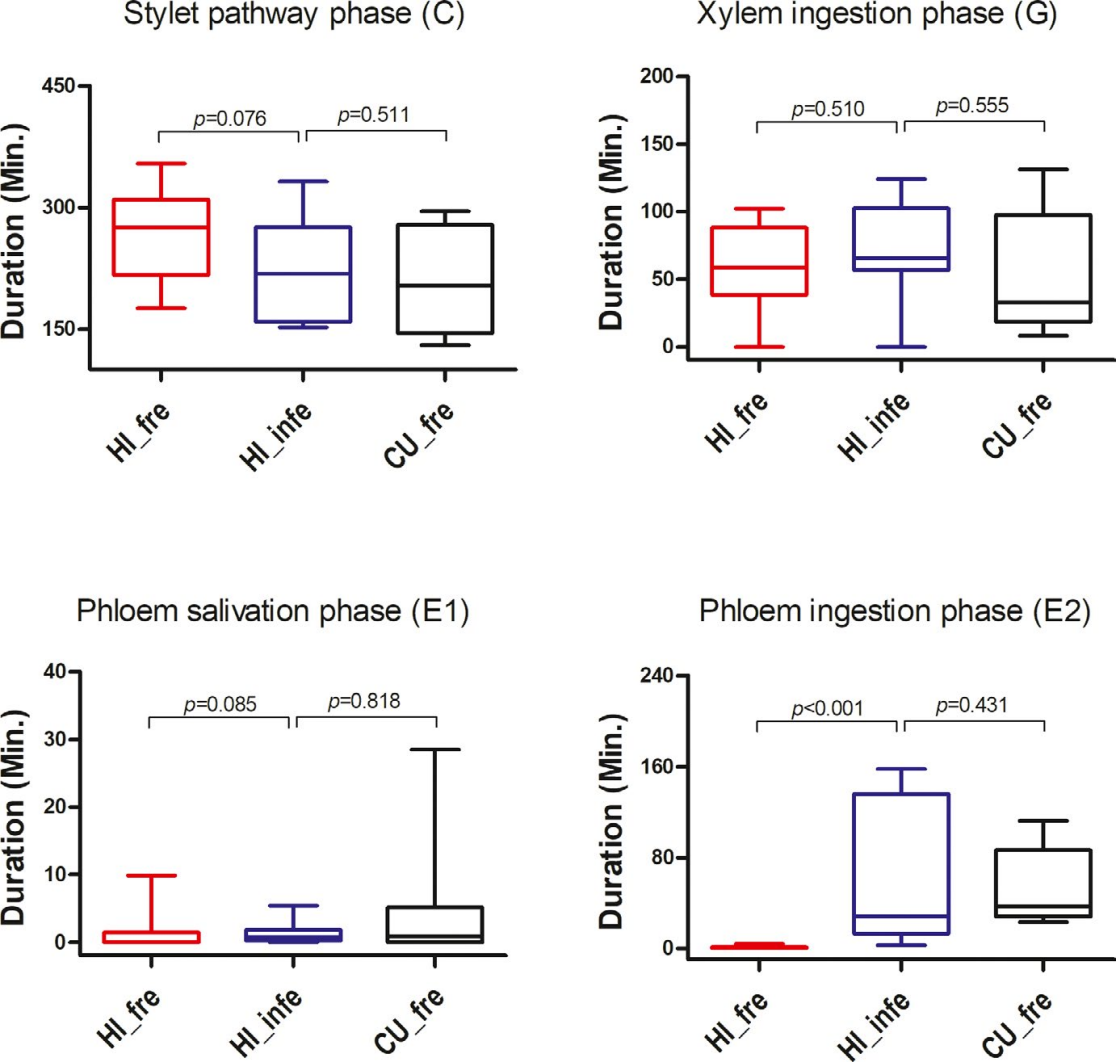
Piercing and sucking behaviors of the hibiscus‐specialized lineage (HI) of *Aphis gossypii* on fresh (fre) and pre‐infected (infe) cucumber leaves with *Pseudoperonospora cubensis*. Vertical whiskers attached to the boxes indicate min. and max. values; means were pairwise compared by Mann–Whitney *U*‐test; the number of repeated individuals: *N*_HI_fre_ = 18, *N*_HI_infe_ = 11 and *N*_CU_fre_ = 11

The duration of the C and E1 waveforms of the HI lineage on pre‐infected cucumber leaves was not different from that on fresh cucumber leaves (Mann–Whitney *U*‐test, *p* = .076 and 0.085, respectively) (Figure 5). However, the duration of the E2 waveform of the HI lineage on pre‐infected cucumber was significantly longer than that on fresh cucumber (*p* < .001), but not different from that of the CU lineage on fresh cucumber (Mann–Whitney *U*‐test, *p* = .431). The duration of the G waveform of the HI lineage on pre‐infected cucumber was not significantly different from that on the fresh cucumber (Mann–Whitney *U*‐test, *p* = .510), neither different from that of the CU lineage on fresh cucumber leaves (Mann–Whitney *U*‐test, *p* = .555). The E2 waveform represents the phase of phloem sap ingestion. The E2 waveform was detected in the HI lineage cultured on pre‐infected cucumber, but not in the HI lineage cultured on fresh cucumber, which indicated that the HI lineage fed successful on pre‐infected cucumber. This result explains why the HI lineage performed much better on pre‐infected cucumber than on fresh cucumber.

## 4 DISCUSSION

**FIGURE 6:**
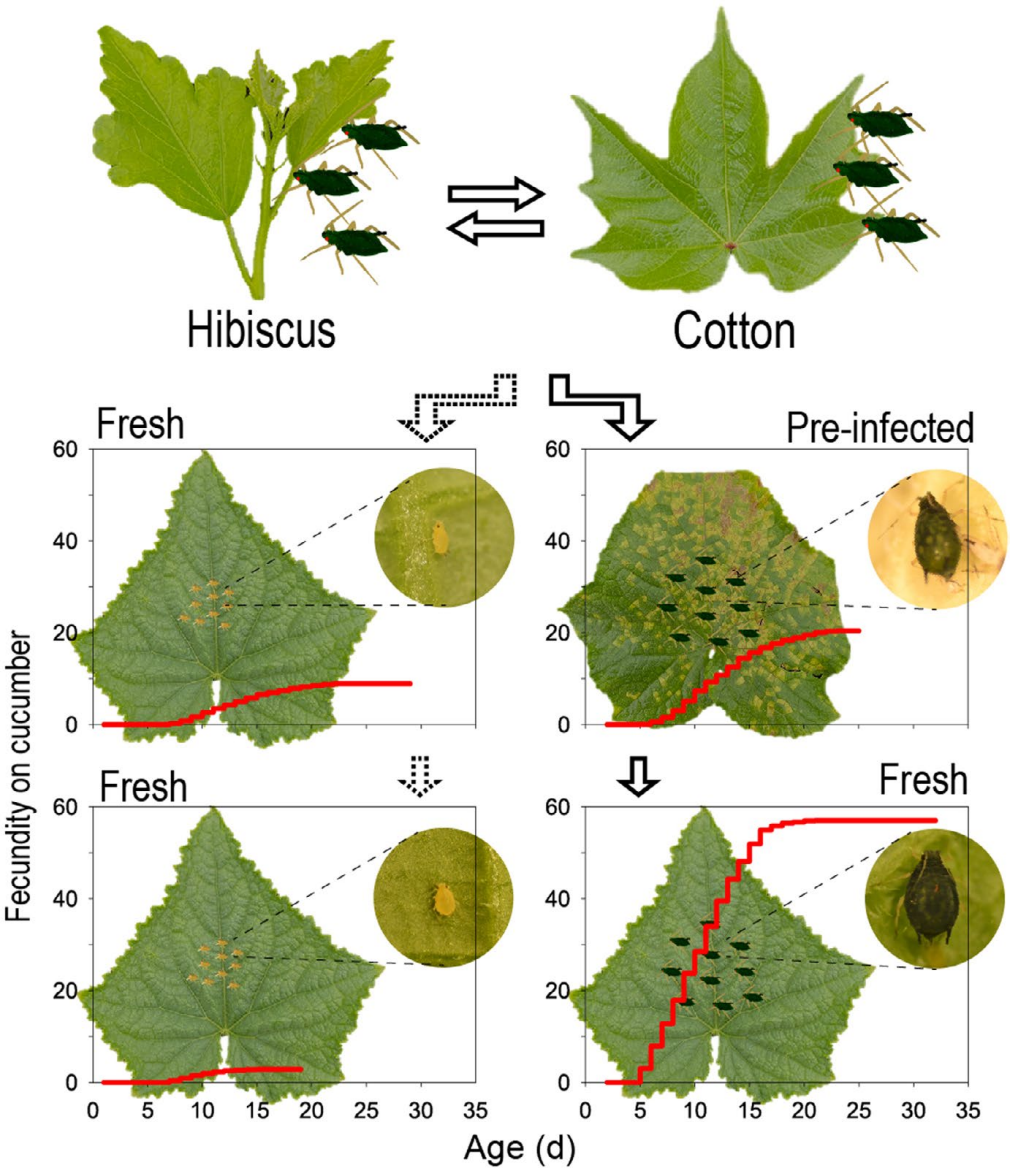
Phytopathogen‐mediated rapid acclimation of the hibiscus‐specialized *Aphis gossypii* to cucumber. The hibiscus‐specialized *A*. *gossypii* cannot use cucumber as host plant but performed well on cucumber that has been pre‐infected with the phytopathogen *Pseudoperonospora cubensis*; a 2‐week feeding experience on the pre‐infected cucumber enables the hibiscus‐specialized *A*. *gossypii* to fully acclimate to fresh cucumber

Our results demonstrated that the host‐specialized lineage of *A*. *gossypii* rapidly acclimated to a new host plant under the assistance of a phytopathogen. Specifically, hibiscus‐specialized *A*. *gossypii* could not use cucumber as a host species, but performed very well on cucumber that was pre‐infected with a biotrophic phytopathogen *P*. *cubensis*, and a short feeding experience on pre‐infected cucumber gave the hibiscus‐specialized lineage of *A*. *gossypii* the ability to fully use intact cucumber as a host plant. This is a new pathway for host‐specialized *A*. *gossypii* to rapidly expand the diet breadth to new host plants (demonstrated in Figure 6). Under field conditions, some cucumber plants are likely to be infected by *P*. *cubensis* that causes the predominant cucumber disease downy mildew. Therefore, it is possible for the hibiscus‐specialized lineage of *A*. *gossypii* to shift to use cucumber via the pre‐infected cucumber.

The cucumber‐ and hibiscus‐specialized (or cotton‐specialized) lineages of *A*. *gossypii* are the most studied host‐specialized populations in this species. Liu et al. (2002) reported that nymphs of *A*. *gossypii* from cotton or hibiscus survived for less than 6 days when transferred to fresh cucumber, and nymphs from cucumber also survived for less than 6 days on cotton; however, nymphs from cotton could use hibiscus as host plant and vice versa. A study from Australia indicated that alate adults from cotton produced significantly fewer nymphs on cucumber or pumpkin than on cotton or hibiscus, and those nymphs could not develop to maturity; alate adults from pumpkin also produced significantly fewer nymphs on cotton or hibiscus than on cucumber or pumpkin (Najar‐Rodríguez et al., 2009). Fitness trade‐offs in host‐associated populations of *A*. *gossypii* have also been reported in European (Carletto et al., 2009; Satar et al., 2013) and Middle Eastern populations of *A*. *gossypii* (Razmjou et al., 2010). The results of this study are in line with those studies. Although the HI lineage in our study did not die out within a few days after being transferred to fresh cucumber, it maintained very low population growth and the nymphs developed into yellow dwarfs. *Aphis gossypii* develops into yellow dwarfs when the population density is high, even on native hosts (Watt & Hales, 1996). However, the yellow dwarfs we observed were different from those dwarfs under high population density because they produced very few offspring and did not inflict damage symptoms on cucumber plants. The poor performance of the HI lineage on fresh cucumber did not improve with the extension of exposure time to fresh cucumber. Thus, the HI lineages used in the present study were highly specialized on their native host.

Genetic adaptation and transcriptional plasticity have been proposed to explain how polyphagous insect herbivores adapt to diverse host plants (Birbaum & Abbot, 2020; Mathers et al., 2017). Genetically distinct host‐associated populations have been frequently reported in aphid species. For example, more than 10 host races with genetic differentiation have been detected in populations of the pea aphid *Acyrthosiphon pisum* in Western Europe (Peccoud et al., 2009). The cotton‐melon aphid *A*. *gossypii* collected from different plant families at large geographical scales have different genetic structures according to host species (Carletto et al., 2009). In the sugarcane aphid, *Melanaphis sacchari*, host transfer experiments demonstrated the existence of fitness trade‐offs, and genetic testing revealed a genetic structure linked to host plants (Nibouche et al., 2015). In *Myzus persicae*, microsatellite DNA analysis revealed genetic divergence between host‐associated populations (Margaritopoulos et al., 2007; Nikolakakis et al., 2003). These populations are genetically differentiated and adapted to their respective host species; hence, they have likely been associated with their host for a long time. However, a recent study showed that *M*. *persicae* could adapt to diverse host plants through transcriptional plasticity of gene expression (Mathers et al., 2017). In our study, the HI lineage of *A*. *gossypii* was highly specialized on hibiscus and cotton; however, feeding experience of as short as 2 weeks (two generations) on the pre‐infected cucumber made the HI lineage fully acclimated to cucumber. Similarly, feeding experience changing host specialization of *A*. *gossypii* was also reported by Ma et al. (2019), who found that feeding experience on an artificial diet led some lineages of the cotton‐specialized and the cucumber‐specialized lineage of *A*. *gossypii* to acquire the ability to use cucumber and cotton, respectively. We think that the rapid acclimation of the HI lineage to cucumber might be related to transcriptional plasticity rather than genetic adaptation. Some genes enabling the use of specific host species may be activated during feeding on pre‐infected cucumber or an artificial diet. We suggested to find these genes controlling specific host utilization by comparing transcriptome or/and proteome of the hibiscus‐specialized lineage before and after acclimation to cucumber, which will lead to a deeper understanding of aphid host specialization.

During feeding process, aphids secrete saliva that contains a range of salivary proteins in plants (Will et al., 2007). Some of these proteins act as effectors that inhibit plant defenses, such as sieve tube occlusion by forisomes; however, plants can recognize certain salivary proteins and then elicit defense responses (Elzinga et al., 2014; Guo et al., 2020; Yates‐Stewart et al., 2020). The balance between defense and counter‐defense determines the host range of specific aphid species (Boulain et al., 2019; Rodriguez & Bos, 2013). Like aphids, phytopathogens also secrete effector proteins or metabolites into plants during infection to manipulate plant defenses (Koeck et al., 2011; Oliva et al., 2010). It is possible that effectors from insect herbivores and plant pathogens converge on the same host plant (Liu et al., 2020). A host‐specialized aphid cannot colonize a non‐native host plant probably because it is short of specific effectors targeting the non‐native plant (Boulain et al., 2019; Rodriguez & Bos, 2013). Theoretically, if a plant has been infested by its compatible pathogens, an incompatible aphid of this plant should be able to feed on it. Our study demonstrated the existence of this possibility. Our EPG data revealed that the HI lineage could not ingest phloem sap on fresh cucumber because no E2 waveform was found, but it could ingest phloem sap on pre‐infected cucumber. The infection of *P*. *cubensis* suppressed the defense responses of cucumber, which probably facilitated subsequent phloem sap ingestion of the HI lineage. We inferred that the HI lineage of *A*. *gossypii* was ‘hitchhiked’ by *P*. *cubensis* in colonizing pre‐infected cucumber. Other phytopathogens (especially biotrophic phytopathogens) may also have the same effects as *P*. *cumbensis* in helping the HI lineage to feed on cucumber. This hypothesis remains to be verified.

## 5 CONCLUSIONS

The present study revealed that the HI lineage of *A*. *gossypii* could colonize cucumber that were pre‐infected with a phytopathogen, and that a short feeding experience on the pre‐infected cucumber enabled the aphid to fully use intact cucumber as a host plant. These results demonstrated that the HI lineage of *A*. *gossypii* can shift from the primary host hibiscus to cucumber under the assistance of the phytopathogen. However, we cannot conclude that *A*. *gossypii* infesting cucumber come from the HI lineage in the field because the CU lineage co‐exists with the HI lineage and *A*. *gossypii* has other primary hosts in the sampling area. Population genetic analyses would help to assess whether *A*. *gossypii* from hibiscus also infest cucumber in the field. Finally, comparative analyses of salivary gland transcriptome and saliva proteome of the HI lineages before and after the switch to cucumber should help to identify activated effector genes that could be responsible for rapid acclimation to a new host species.

## CONFLICT OF INTEREST

We declare that we have no conflict of interest.

## AUTHOR CONTRIBUTION

**Farhan Ali:** Data curation (lead); Formal analysis (supporting); Software (lead); Writing‐original draft (lead); Writing‐review & editing (supporting). **Xiaoyue Hu:** Data curation (supporting); Investigation (supporting); Writing‐review & editing (supporting). **Duoqi Wang:** Data curation (supporting); Investigation (supporting); Software (supporting); Writing‐review & editing (supporting). **Fengying Yang:** Data curation (supporting); Methodology (supporting); Software (supporting). **Hao Guo:** Data curation (supporting); Writing‐review & editing (supporting). **Yongmo Wang:** Conceptualization (lead); Formal analysis (lead); Investigation (lead); Methodology (lead); Supervision (lead); Writing‐review & editing (lead).

## Supporting information

Figure S1

## Data Availability

Data available via the Dryad Digital Repository at https://doi.org/10.5061/dryad.pvmcvdnmv.
