# Supplementary figures and images for: Plant pathogen‐mediated rapid acclimation of a host‐specialized aphid to a non‐host plant
Source: Ecol Evol. 2021 Oct 11;11(21):15261–72. doi: 10.1002/ece3.8209 (PMC8571567; doi:10.1002/ece3.8209)

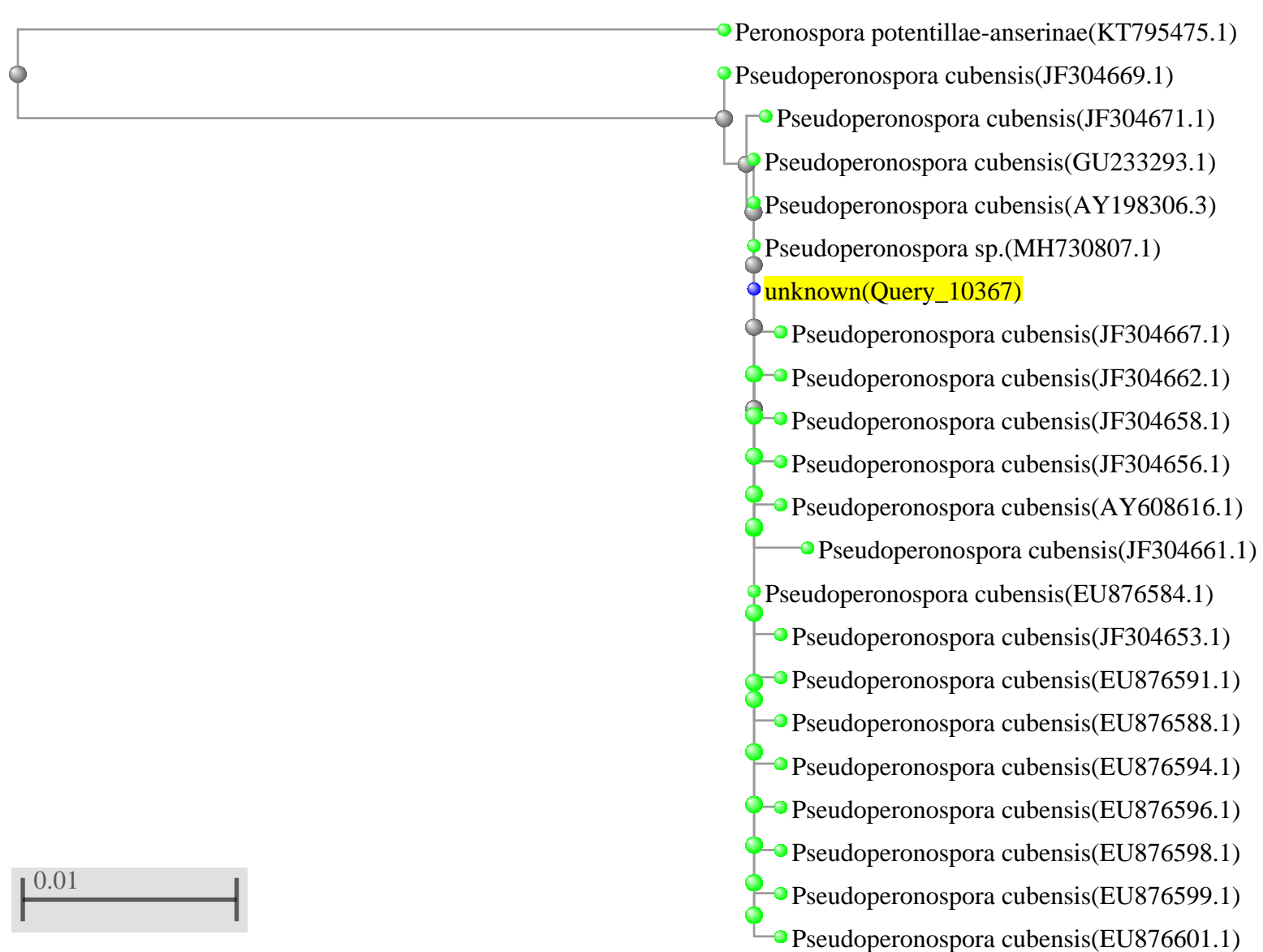

Supplement: Supplementary file 1 — Figure S1 [file ECE3-11-15261-s001.pdf]
